# Cognitive Impairment, Depression, and Cooccurrence of Both among the Elderly in Panama: Differential Associations with Multimorbidity and Functional Limitations

**DOI:** 10.1155/2015/718701

**Published:** 2015-12-21

**Authors:** Alcibiades E. Villarreal, Shantal Grajales, Lineth Lopez, Gabrielle B. Britton, Panama Aging Research Initiative

**Affiliations:** ^1^Centro de Neurociencias y Unidad de Investigación Clínica, Instituto de Investigaciones Científicas y Servicios de Alta Tecnología (INDICASAT AIP), Ciudad del Saber, Panama; ^2^Department of Biotechnology, Acharya Nagarjuna University, Guntur 522 510, India; ^3^Department of Hematology, Complejo Hospitalario Dr. Arnulfo Arias Madrid de la Caja de Seguro Social, Panama City, Panama

## Abstract

Cognitive impairment and depression are common mental health problems among the elderly, although few studies have examined their cooccurrence in older adults in Latin America. The purpose of this study was to examine cognitive impairment, depression, and cooccurrence of the two conditions and associated factors in a sample of older adults in Panama. This study included 304 community-dwelling elderly (≥65 years) individuals. Participants underwent a clinical interview and assessments of cognitive function by the Minimental State Examination and depressive symptoms by the Geriatric Depression Scale. Limitations in basic (BADL) and instrumental (IADL) activities in daily living and the presence of chronic illnesses were recorded. Multinomial regression analysis revealed that cooccurrence of cognitive impairment and depressive symptoms was explained by increasing age (OR: 3.2, 95% CI: 1.20, 8.30), low education (OR: 3.3, 95% CI: 1.33, 8.38), having four or more chronic conditions (OR: 11.5, 95% CI: 2.84, 46.63), and BADL limitations (OR: 5.0, 95% CI: 1.26, 19.68). Less education and limitations in BADL and IADL increased the odds of cognitive impairment alone, while less education and three or more chronic conditions increased the odds of depression alone. These findings underscore the relevance of assessing cognitive impairment in the elderly as part of a long-term approach to managing depression and vice versa.

## 1. Introduction

Latin America and Caribbean (LAC) region is experiencing one of the fastest rates of population aging [1]. Population-based studies confirm that rates of common age-related chronic illnesses such as dementia and mild cognitive impairment (MCI) are similar to those of developed countries [2, 3], and cross-sectional surveys from a multicenter cohort [4] revealed high prevalence of depression (21.5–33.2%) across six cities in the region [5]. These studies reveal concomitant rates of modifiable risk factors for depression and cognitive impairment such as diabetes, cardiovascular disease, and illiteracy. Consequently, the burden of age-related disorders is expected to be especially high in LAC countries in the coming decades.

Older adults are disproportionately affected by several chronic conditions. Among the elderly, chronic conditions such as cognitive impairment and depression are interrelated and often coexist [6–9], and at least one report confirms that each condition alone contributes to the increase of an older person's risk for mortality [10]. Recent studies have shown that subjects with MCI present more depressive symptoms compared with those without cognitive impairment [7, 11, 12]. Additionally, in a study of community-dwelling adults in Spain aged 65 years and older, the coexistence of cognitive impairment and depression was found to be associated with chronic illnesses and impairment in activities of daily living [13]. In that study, the primary medical conditions associated with coexisting cognitive impairment and depression included dementia, congestive heart failure, cerebrovascular disease, and diabetes, most of which were distinct from those associated with cognitive impairment alone or depression alone [13]. In elderly subjects, the convergence of depressive symptoms with other chronic conditions may represent a barrier to diagnosis and subsequent treatment of depression [14]. Taken together, these results suggest that, in the geriatric population with cognitive decline, depression, or both conditions coexisting, individuals should be screened for other conditions such as chronic diseases [13, 15].

Studies have shown that depression is associated also with functional limitations during aging, and this relationship is influenced by physical limitations that arise as a product of chronic illness comorbidity, namely, stroke, respiratory problems, cancer, and diabetes [14, 16]. Besides depression and comorbidities, functional disability can be significantly influenced by cognitive impairment. An early study demonstrated that cognitive status can be a predictor of functional status independently of whether individuals present psychiatric disorders [17]. Since then, longitudinal studies have shown that difficulties in the performance of activities of daily living are greater in subjects with cognitive impairment, and, further, cognitive decline is correlated with declines in activities of daily living performance over time [18]. More studies are needed to establish the combined effects of cognitive decline and depression on chronic conditions and limitations in activities of daily living.

In the present study, we conducted the first examination of depressive symptoms, cognitive impairment, or both conditions coexisting and their association with functional disability and multimorbidity in a sample of elderly individuals in Panama, an upper middle-income country in the LAC region. Although Panama is advancing toward an aged society [19], there is lack of research focused on age-related chronic conditions [20]. Age-related health problems are complicated by high rates of poverty, low education, and lack of access to health care, which affect vulnerable populations such as the elderly disproportionately. Also, systematic screening of cognitive function and depression is lacking in primary and community-based health care for adults despite evidence of its utility in predicting mortality [10]. Therefore, research regarding potentially modifiable risk factors for cognitive impairment and depressive symptoms in older adults could be important for developing effective geriatric health care public initiatives. Based on previous reports [7, 13], we hypothesized that cognitive function and depressive symptoms would be associated independently and in combination with functional disability and chronic illness comorbidity.

## 2. Methods

### 2.1. Participants

Data from this study came from the Panama Aging Research Initiative (PARI) study, the first-ever study of Panamanian aging. PARI participants were recruited from the outpatient geriatric services of the largest public hospital of the Social Security (CSS) located in Panama, the capital city of Panama. Inclusion criteria included being 65 years or older, willingness to participate in the baseline interview and three follow-up visits over the course of 12–18 months, and provision of informed consent. Exclusion criteria included any medical condition that required hospitalization and participation in an ongoing clinical study at the time of enrollment.

The present report constitutes an analysis of the data collected during the baseline interview and 3-to-6-month (M = 4.5 months, SD = 1.9) follow-up assessment of cognitive function and depressive symptoms. At baseline, each participant underwent a physical exam and clinical interview and responded to items regarding demographic factors, medical conditions, and functional status. Interviewers included physicians, medical students, and graduate students. In total, 423 participants were enrolled and 326 community-dwelling (noninstitutionalized) persons completed the baseline interview and follow-up assessments of cognitive function and depressive symptoms. Of these, 15 participants were excluded because they were illiterate and seven were excluded due to serious mental or physical disabilities. The present report includes data from 304 participants.

An analysis comparing the 97 participants who did not return to complete the follow-up assessment with those who did revealed that a greater proportion of those who did not return were older (80+ years of age, 58.9% versus 43.9%) and more likely to have at least one BADL (71.6% versus 52.1%) and at least one IADL (81.1% versus 68.0%) limitation than those who returned.

### 2.2. Ethics Statement

The study protocol was approved by the National Bioethics Committee of the Instituto Conmemorativo Gorgas de Estudios de la Salud and the Institutional Bioethics Committee of the CSS. Each participant (or informant/caregiver) signed informed consent forms and patient confidentiality was not breached in accordance with the Declaration of Helsinki (1964).

### 2.3. Variables and Instruments

Participants underwent an interview, physical exam, clinical interview, and nonfasting blood draw. The 30-item Spanish version of the Minimental State Examination (MMSE) was used as a measure of global cognition [21]. The reverse spelling of the word “world” in the attention item was used instead of the backward serial sevens. MMSE scores were adjusted for age and level of education [22]. Two categories were defined using the MMSE test scores: cognitively impaired (<24) and unimpaired (≥24). Depressive symptoms were assessed with the Spanish version of the 30-item Geriatric Depression Scale (GDS-30) [23, 24]. The instrument was applied by the investigator reading the items out loud and asking the participants to respond to each of the items. A cut-off score of ≥11 on the GDS was used to classify depressed individuals. Thus, in this report, the term* depression* is used to describe those participants who scored ≥11 on the GDS.

Chronic conditions were recorded through self-report and were assessed by answers to questions regarding physician diagnosis and current medications. Subjects were asked, “Has a doctor or nurse ever told you that you had…?” The following conditions were assessed: hypertension, coronary heart disease, diabetes, stroke, cancer, chronic lung disease, and arthritis. The number of chronic diseases was reported as a categorical disease indicator of whether a participant had at least one, two, three, or four or more conditions (the smallest two categories were grouped due to small numbers). Participants were asked also to report whether they smoked currently or had ever smoked, and responses were dichotomized as “current/past smoker” versus “never smoked.”

Performance in activities of daily living was evaluated through self-report. Subjects were asked to indicate whether they had any difficulty performing the following seven BADL: transferring, bathing, dressing, grooming, toileting, feeding, and continence. Likewise, disability in seven IADL was evaluated: leaving the home independently in public or private transportation, preparing a meal, using a telephone, grocery shopping, performing basic house chores (housekeeping, laundry), handling money, and taking medications. IADL scores were corrected in cases where the individual had never performed a task. A score of zero (0) was assigned when the subject was able to perform the task without difficulty; a score of one (1) was assigned when the subject was able to perform the task with difficulty or was unable to perform the task. Limitations in BADL and IADL were dichotomized into “none” versus “at least one.”

### 2.4. Statistical Analysis

Analyses were performed using SPSS 21.0 statistical software. Descriptive statistics (frequency and percentage) were computed for all variables across groups and categorical differences were examined using chi square analysis. We applied multinomial logistic regression to identify factors associated with three outcomes, cognitive impairment alone, depression alone, and coexisting cognitive impairment and depression, using the absence of cognitive impairment and depression as the reference category. Odds ratios (OR) and 95% confidence intervals (95% CI) are presented in each case. Statistical significance was set at *p* < .05.

## 3. Results

### 3.1. Demographics of the Sample

Table 1 summarizes the distribution of cognitive impairment, depression, and cooccurrence of the two in the study sample. According to the MMSE performance, 21.4% (95% CI: 17.1, 26.3) of participants were found to have cognitive impairment without depression and 11.2% (95% CI: 8.1, 15.2) were classified as having both cognitive impairment and depression. According to the GDS, 18.1% (95% CI: 14.2, 22.8) of participants were found to have depression without cognitive impairment. Notably, almost half (49.3%; 95% CI: 43.8, 54.9) of participants were classified as having neither cognitive impairment nor depression. Average MMSE score was 23.9 (SD = 5.6) and average GDS score was 8.0 (SD = 5.7). MMSE and GDS scores were significantly correlated (*r* = −0.13, *p* = .024), indicating that better cognitive function was associated with less depression symptomatology.

In this study, we evaluated the demographic factors, number of chronic conditions, and presence of functional limitations as a function of cognitive status, depression, and the cooccurrence of both conditions (Table 2). Participant ages ranged from 65 to 102 years with a mean age of 78.2 (SD = 7.5). Approximately 66% of participants were female (*n* = 200) and approximately half the sample (52.6%) completed primary education or less. Participants reported an average of 1.9 (SD = 1.0) chronic illnesses. The oldest participants (≥80 years) presented more cognitive impairment and cooccurring cognitive impairment and depression than younger participants. Less educated participants also presented more cognitive impairment, depression, and cooccurring cognitive impairment and depression than participants whose schooling extended beyond primary school. Participants with four chronic conditions or more presented more cooccurring depression and cognitive impairment and depression alone than those with fewer chronic conditions. With regard to functional limitations, participants with at least one BADL limitation or one IADL limitation were more likely to present cognitive impairment alone or cooccurring cognitive impairment and depression than those with no limitations.

### 3.2. Factors Associated with Cognitive Impairment, Depressive Symptoms, and Both Conditions Coexisting

Multinomial logistic regression analyses confirmed significant associations of age, education, number of chronic conditions, and BADL limitations with cooccurring cognitive impairment and depression (Table 3). The oldest participants (≥80 years) were 3.2 times more likely (95% CI: 1.20, 8.30) to be classified as cognitively impaired and depressed as younger participants. Likewise, those with less schooling (≤6 years) were 3.3 times more likely (95% CI: 1.33, 8.38) to be cognitively impaired and depressed compared to those whose schooling extended beyond primary school. In addition, having four or more chronic illnesses (OR: 11.5, 95% CI: 2.84, 46.63) and at least one BADL limitation (OR: 5.0, 95% CI: 1.26, 19.68) was associated with greater likelihood of cooccurring cognitive impairment and depression. After fitting the multinomial logistic regression, lower education levels remained significantly associated with cognitive impairment alone (OR: 2.8, 95% CI: 1.39, 5.72) and depression alone (OR: 2.4, 95% CI: 1.13, 4.98), but age was not associated with either condition alone. Lastly, functional limitations in at least one BADL or IADL remained significantly associated with cognitive impairment (*p*s < .02) but only marginally significant for depression alone. Suffering three or more chronic conditions was significantly associated with depression (*p*s < .003), but multimorbidity was not associated with cognitive impairment alone. Gender, marital status, and smoking status were not significantly associated with any condition.

## 4. Discussion

In the present study, we assessed cognitive impairment, depression, and cooccurrence of the two conditions and related factors in subjects aged 65 and older. Significant associations were observed between education and BADL and IADL limitations and cognitive impairment alone, while educational level and multimorbidity were associated with depression alone. The factors specifically related to coexisting cognitive impairment and depression were low education, having four or more chronic illnesses, and having at least one limitation in BADL. These results are consistent with previous studies that show that poor cognitive function and depressive symptoms cooccur among the community-dwelling elderly and are associated with chronic illnesses and impairment in activities of daily living [6, 7, 13]. Although we did not examine the association between cognitive impairment and depression and individual chronic illnesses, we showed that suffering three or more chronic illnesses was associated with the greatest likelihood of depression alone whereas suffering four or more chronic illnesses was associated with coexisting cognitive impairment and depression. In contrast, no significant association was found between chronic illnesses and cognitive impairment alone. Depression in the elderly has been shown to be associated with nonpsychiatric hospitalization, longer length of hospital stay, and higher mortality [25–27]. Importantly, the combined impact of cognitive impairment and depression has been shown to increase the risk for mortality relative to either condition alone [10].

Our results also confirm that depressive symptomatology is correlated with cognitive impairment. In a study in Japanese individuals aged 65 years and older, cognitive impairment was more prevalent in individuals with depression, and, conversely, individuals with mild cognitive impairment were more likely to develop depression [7]. Likewise, in a population-based study of elderly individuals aged 60 years and older in Mexico examining cognitive impairment and depression, assessed with the MMSE and GDS, respectively, cognitive impairment was associated with depression as well as with being older than 75 years, being unmarried, and having less education [6]. In the same study, depression was associated with the same factors as cognitive impairment in addition to being female, but the study did not examine the factors associated with the combination of cognitive impairment and depression. Although most studies in Latin America have found that women are marginally more at risk for cognitive impairment and depression, we did not find associations between cognitive impairment or depression and gender. However, low educational achievement, which is often linked to poverty or lower socioeconomic status, showed a strong association with cognitive impairment, depression, and the cooccurrence of both conditions. These results are consistent with reports of associations between low levels of education with poorer mental health and increased risk of disease comorbidity [28] and suggest that individuals in Panama whose studies extend beyond primary school are more likely to age in better health. This finding is particularly relevant in Panama where the average educational level is 9.2 years [29].

Numerous studies have confirmed an association between increasing depression and disability [7, 13, 30, 31]. Disability in the elderly, characterized by the loss of ability to perform activities of daily living, is associated with significant burdens, including increased risk of hospitalization, institutionalization, and mortality [32, 33]. Moreover, different chronic conditions such as diabetes, mild cognitive impairment, dementia, and cerebrovascular events, such as stroke, affect the elderly disproportionately relative to the other conditions [13, 34]. Population-based studies in seven Latin American cities indicate that the proportion of community-dwelling adults aged 60 years and older reporting any BADL or IADL disability is approximately 19%, and 44% had more than one chronic condition [30], a finding which underscores the burden of disease and disability in the region.

An important limitation of the present study is the nature of the sample (outpatient-based) and the selection bias that resulted from the loss of older and more impaired subjects, and thus our results most likely underestimate the extent of cognitive impairment and depression in the elderly population of Panama. Another limitation is that self-reported disability and chronic illnesses may be affected by sociocultural factors, and so comparisons of our results with those of other LAC countries should be made with caution. Although evidence suggests that self-report provides accurate estimates of disability and disease comorbidity [35] and predicts mortality and other clinical health measures [36], recent evidence suggests that individuals of lower socioeconomic status show less reliable self-assessments of health [37]. Lastly, our data were obtained over a short time span and do not address the relationship between multimorbidity and progression of cognitive decline and depressive symptoms. Each of these limitations is being addressed in ongoing studies. Study strengths include providing the first report of cognitive impairment and depression in the elderly in Panama, as well as the use of detailed clinical interviews to record the presence of chronic illnesses and other patient information and the use of multiple items for assessing BADL and IADL.

## 5. Conclusions

Previous studies have shown that in late life coexisting depression and cognitive impairment may contribute to an elderly person's vulnerability. To our knowledge, ours is the first report from Panama of coexisting cognitive impairment and depressive symptomatology in community-dwelling adults of any age. Our results add to the existing knowledge regarding the presence of cognitive impairment and geriatric depression and their associated clinical factors, namely, multimorbidity and limitations in activities of daily living. Importantly, cooccurrence of cognitive impairment and depression complicates treatment in the elderly, and thus assessments of cognitive impairment in this population should be part of a long-term approach to managing depression and vice versa. The current study supports the hypothesis that multimorbidity particularly affects elderly individuals with depression alone and with coexisting depression and cognitive impairment and sets the stage for additional studies examining the long-term outcomes in follow-up studies.

## Figures and Tables

**Table 1:** Frequency of cognitive impairment, depressive symptoms, and the cooccurrence of both.

	No depressive symptoms (GDS < 11)	Depressive symptoms (GDS ≥ 11)	Total
	Number (%) 95% CI	Number (%) 95% CI	Number (%) 95% CI
No cognitive impairment (MMSE ≥ 24)	150 (49.3) 43.8, 54.9	55 (18.1) 14.2, 22.8	205 (67.4) 62.0, 72.5

Cognitive impairment (MMSE < 24)	65 (21.4) 17.1, 26.3	34 (11.2) 8.1, 15.2	99 (32.6) 27.6, 38.0

Total	215 (70.7) 65.4, 75.6	89 (29.3) 24.5, 34.6	304 (100%)

GDS: Geriatric Depression Scale (30-item); MMSE: Minimental State Examination.

**Table 2:** Comparisons of sociodemographic factors, multimorbidity, and limitations in BADL and IADL among participants with neither cognitive impairment nor depression (*n* = 150), cognitive impairment only (*n* = 65), depression only (*n* = 55), and cooccurring cognitive impairment and depression (*n* = 34).

	Neither cognitive impairment nor depression	Cognitive impairment	Depression	Cooccurring cognitive impairment and depression	*χ* ^2^	*p*
	Number (%)	Number (%)	Number (%)	Number (%)		
Gender						
Male	60 (57.7)	20 (19.2)	14 (13.5)	10 (9.6)	4.80	.187
Female	90 (45.0)	45 (22.5)	41 (20.5)	24 (12.0)		
Age						
65–79 years	98 (55.7)	31 (17.6)	38 (21.6)	9 (5.1)	22.78	<.001
80+ years	52 (40.6)	34 (26.6)	17 (13.3)	25 (19.5)		
Marital status						
Widowed/single/divorced	72 (47.1)	35 (22.9)	26 (17.0)	20 (13.1)	1.83	.608
Married/partnered	78 (51.7)	30 (19.9)	29 (19.2)	14 (9.3)		
Education						
≤6 years	60 (37.5)	45 (28.1)	32 (20.0)	23 (14.4)	20.54	<.001
>6 years	90 (62.5)	20 (13.9)	23 (16.0)	11 (7.6)		
Smoking						
Never smoked	102 (49.3)	47 (22.7)	36 (17.4)	22 (10.6)	0.88	.828
Current/past smoker	48 (49.5)	18 (18.6)	19 (19.6)	12 (12.4)		
Chronic conditions						
0-1	47 (61.0)	24 (31.2)	2 (2.6)	4 (5.2)	69.86	<.001
2	60 (60.0)	21 (21.0)	12 (12.0)	7 (7.0)		
3	31 (43.7)	15 (21.1)	16 (22.5)	9 (12.7)		
4+	12 (21.4)	5 (8.9)	25 (44.6)	14 (25.0)		
BADL limitations						
None	76 (75.2)	4 (4.0)	18 (17.8)	3 (3.0)	51.17	<.001
At least one	74 (36.5)	61 (30.0)	37 (18.2)	31 (15.3)		
IADL limitations						
None	70 (66.0)	6 (5.7)	26 (24.5)	4 (3.8)	39.72	<.001
At least one	80 (40.4)	59 (29.8)	29 (14.6)	30 (15.2)		

BADL: basic activities of daily living; IADL: instrumental activities of daily living.

**Table 3:** Multinomial logistic regression model predicting the effect of functional limitations and multimorbidity on cognitive impairment, depression, and both of them coexisting, adjusting for sociodemographic factors and smoking history.

Characteristic	Cognitive impairment (*n* = 65)		Depression (*n* = 55)		Cognitive impairment and depression (*n* = 34)	
	OR (95% CI)	*p*	OR (95% CI)	*p*	OR (95% CI)	*p*
Gender						
Female	1.0 (.43, 2.48)	.941	1.9 (.74, 4.82)	.184	1.4 (.46, 4.56)	.533
Male	—		—		—	
Age						
80+ years	1.1 (.52, 2.13)	.879	.9 (.40, 2.04)	.804	3.2 (1.20, 8.30)	.020
65–79 years	—		—		—	
Marital status						
Widowed/single/divorced	1.4 (.66, 2.97)	.381	1.1 (.50, 2.49)	.782	1.9 (.69, 5.15)	.214
Married/partnered	—		—		—	
Education						
≤6 years	2.8 (1.39, 5.72)	.004	2.4 (1.13, 4.98)	.022	3.3 (1.33, 8.38)	.010
>6 years	—		—		—	
Smoking						
Current/past smoker	.7 (.30, 1.55)	.354	1.1 (.47, 2.43)	.877	1.0 (.38, 2.80)	.962
Never smoked	—		—		—	
Chronic conditions						
0-1	—		—		—	
2	.6 (.26, 1.42)	.252	4.5 (.93, 21.43)	.062	1.0 (.26, 4.17)	.950
3	.6 (.25, 1.55)	.307	11.8 (2.43, 56.81)	.002	2.3 (.59, 8.77)	.235
4+	.6 (.17, 2.04)	.396	53.2 (10.54, 268.19)	<.001	11.5 (2.84, 46.63)	.001
BADL limitations						
At least one	9.6 (3.07, 29.74)	<.001	2.3 (.95, 5.42)	.064	5.0 (1.26, 19.68)	.022
None	—		—		—	
IADL limitations						
At least one	3.4 (1.25, 9.53)	.017	.4 (.17, 1.05)	.063	1.4 (.39, 5.26)	.593
None	—		—		—	
